# From resistance mechanisms to therapy: Antimicrobial resistance in Gram-negative bacteria

**DOI:** 10.71150/jm.2604017

**Published:** 2026-08-06

**Authors:** Minho Lee

**Affiliations:** 1 Department of Microbiology, College of Medicine, Hallym University, Chuncheon 24252, Republic of Korea; 2 Institute of Medical Science, College of Medicine, Hallym University, Chuncheon 24252, Republic of Korea

**Keywords:** Gram-negative bacteria, antimicrobial resistance, β-lactamases, carbapenemases, efflux pump, therapeutic strategies

## Abstract

Antimicrobial resistance poses a major global health challenge, and infections caused by multidrug-resistant Gram-negative bacteria are associated with substantial morbidity and mortality. In contrast to many Gram-positive pathogens, Gram-negative bacteria combine intrinsic barriers with acquired determinants, including enzymatic drug inactivation, reduced outer membrane permeability, active efflux, and target modifications, which collectively compromise the efficacy of multiple antibiotic classes. Previous reviews have largely catalogued resistant pathogens or antimicrobial agents. This review provides a mechanism-focused overview of antimicrobial resistance in clinically important Gram-negative bacteria and explains how dominant resistance determinants translate into clinically relevant failure modes, such as delayed effective therapy, limited treatment options, and increased reliance on toxic last-line agents. Current and emerging therapeutic strategies are discussed through a mechanism-based lens, emphasizing newer β-lactam/β-lactamase inhibitor combinations and nontraditional approaches, including phages, antivirulence, and microbiome-based interventions. This review highlights the conceptual links between resistance mechanisms, clinical impact, and rational therapeutic choices and identifies priorities for future research aimed at mitigating antimicrobial-resistant Gram-negative infections.

## Introduction

Antimicrobial resistance (AMR) is a major global public health challenge (GBD 2021 Antimicrobial Resistance Collaborators, 2024; Macesic et al., 2025; Miller and Arias, 2024). Global estimates indicate that bacterial AMR was responsible for 1.27 million deaths and associated with 4.95 million deaths in 2019, and the burden remained high in 2021, disproportionately affecting low- and middle-income countries (Antimicrobial Resistance Collaborators, 2022; GBD 2021 Antimicrobial Resistance Collaborators, 2024; Thompson, 2022). Clinically important AMR is concentrated in a limited set of pathogens, often grouped as the ESKAPE organisms (De Oliveira et al., 2020; Rice, 2008). Although this group includes major Gram-positive pathogens such as *Enterococcus faecium* and *Staphylococcus aureus*, the present review focuses on Gram-negative members because they combine envelope-associated intrinsic barriers with highly mobile acquired resistance determinants and account for a substantial proportion of the global AMR burden (Miller and Arias, 2024). Consistent with this focus, the 2024 World Health Organization Bacterial Priority Pathogen List identified multiple multidrug-resistant Gram-negative organisms as critical priorities for research and control (WHO, 2024).

Several recent reviews have provided focused discussions on antimicrobial development platforms that are related to, yet distinct from, the scope of this review. These include peptide-based antimicrobials, antibiotic hybrids, structural and mechanistic aspects of β-lactamase inhibitors, proteostasis-targeted antibacterial strategies, and synthetic biology-assisted approaches such as CRISPR-Cas systems, engineered bacteriophages, microbiome engineering, and metabolic engineering (Jeong et al., 2026; Kim, 2026; Lee et al., 2026; Oh et al., 2026; Park et al., 2026). Building on these platform-focused perspectives, the present review adopts a complementary framework centered on clinically important Gram-negative bacteria, linking recurrent resistance mechanisms to predictable clinical failure modes and mechanism-informed therapeutic decisions.

Multiple factors drive the expansion of AMR in Gram-negative bacteria. These organisms are particularly efficient at disseminating resistance determinants through horizontal gene transfer mechanisms, including transformation, transduction, and conjugation (Peleg and Hooper, 2010). In addition, unlike Gram-positive bacteria, Gram-negative bacteria combine an outer membrane diffusion barrier, variable porin architecture, and efflux capacity, resulting in heterogeneous susceptibility profiles even within the same species (Maher and Hassan, 2023; Oliver et al., 2024; Saxena et al., 2023). Therefore, the practical challenge lies not only in pathogen diversity but also in the ways envelope biology and mobile genetic elements jointly undermine the activity of multiple antibiotic classes, including last-resort agents such as carbapenems and polymyxins, as well as new β-lactam/β-lactamase inhibitor (BL/BLI) combinations (Giddins et al., 2018; Macesic et al., 2020, 2025; Nordmann and Poirel, 2019; Tamma et al., 2024). Clinically, infections caused by AMR Gram-negative pathogens present as common syndromes, including urinary tract, respiratory, intra-abdominal, and bloodstream infections, and the risk is increased by prior antibiotic exposure and healthcare contact (Palacios-Baena et al., 2021; Paul et al., 2022; van Loon et al., 2018). Contemporary guidelines increasingly incorporate risk-stratified empiric and definitive choices; however, bedside decisions remain constrained by delayed susceptibility data and heterogeneous local epidemiology (Paul et al., 2022). Accordingly, this review prioritizes a “mechanism → failure mode → therapeutic option” framework rather than an encyclopedic catalogue of organisms or drugs, aligning resistance determinants with practical decision points, including the use of BL/BLI combinations, escalation to novel agents, and selected nontraditional approaches.

## Core Resistance Mechanisms

Antibiotics target multiple bacterial processes to inhibit growth or induce cell death (Fig. 1). Gram-negative bacteria can resist these agents through antibiotic inactivation or modification, reduced drug entry, active efflux, target modification, and tolerance-associated physiological states (Fig. 2, Table 1) (Bush and Bradford, 2019; Dulanto Chiang and Dekker, 2024; Rocker et al., 2020; Yang and Hu, 2022). These resistance phenotypes do not arise exclusively through chromosomal mutation, but emerge through multiple overlapping routes, including intrinsic envelope-associated barriers, horizontal acquisition of resistance determinants, stable mutations in drug targets or regulatory loci, inducible or derepressed expression of pre-existing resistance systems, and adaptive responses to antimicrobial or envelope stress (Arzanlou et al., 2017; Fernandez and Hancock, 2012; Munita and Arias, 2016). This distinction is especially important for non-enzymatic mechanisms. Efflux pump upregulation and porin repression can occur through regulatory pathways without acquisition of new resistance genes, while redundancy among resistance-nodulation-division (RND)-family efflux systems can amplify pre-existing export capacity under selective pressure (Blair et al., 2014; Li et al., 2015; Tian et al., 2016). In *Pseudomonas aeruginosa* (*P. aeruginosa*), for example, envelope-stress regulation can activate MexAB-OprM expression, whereas OprD repression can reduce carbapenem entry (Ochs et al., 1999; Tian et al., 2016). Thus, mutation is one important route to resistance, but clinically relevant non-susceptibility often reflects the combined effects of acquired determinants, regulatory adaptation, and pre-existing envelope physiology (De Oliveira et al., 2020; Pages et al., 2008). Mobile genetic elements, including plasmids, integrons, and transposons, further accelerate dissemination by carrying determinants that confer resistance to multiple antibiotic classes and facilitate spread across species and clonal lineages (Bhat et al., 2023; Wang et al., 2024).

### β-Lactamase-mediated inactivation

β-Lactamase-mediated hydrolysis remains one of the most clinically important mechanisms of β-lactam resistance in Gram-negative bacteria, but its therapeutic implications depend on enzyme class, inhibitor susceptibility, expression level, and envelope context. A key distinction exists between serine β-lactamases (SBLs; Ambler classes A, C, and D) and metallo-β-lactamases (MBLs; Ambler class B). Through an active-site serine, SBLs hydrolyze β-lactam and are variably inhibited by currently available β-lactamase inhibitors, whereas MBLs require zinc-dependent catalysis and are not directly inhibited by most approved inhibitors (Bush and Bradford, 2019; Park et al., 2026). Because the structural and catalytic features of β-lactamases and their inhibitors have recently been reviewed in detail, the present review focuses on how enzyme class, inhibitor susceptibility, and envelope context translate into clinical failure modes and mechanism-informed treatment choices (Park et al., 2026).

This catalytic distinction underpins the success or failure of specific inhibitor–enzyme pairings. Avibactam inhibits many class A enzymes, including KPC, as well as class C AmpC enzymes and some class D enzymes, including OXA-48-like carbapenemases. Therefore, ceftazidime–avibactam is a rational option when these determinants predominate and susceptibility is retained (Bush and Bradford, 2019; Tamma et al., 2024). In contrast, MBL-producing organisms, such as NDM-, VIM-, or IMP-producing Enterobacterales, create a therapeutic gap because these enzymes hydrolyze carbapenems and many other β-lactams while remaining outside the direct inhibitory spectrum of most approved β-lactamase inhibitors (Boyd et al., 2020; Nordmann and Poirel, 2019; Tamma et al., 2024). In this setting, mechanism matching requires pathway switching rather than simple escalation within carbapenems. Aztreonam-based strategies are biologically rational because aztreonam is stable to MBL-mediated hydrolysis; however, they require protection from co-produced SBLs by an active inhibitor such as avibactam (Boyd et al., 2020; Tamma et al., 2024).

Carbapenem resistance further illustrates why β-lactamase detection must be interpreted within envelope context. KPC production can confer high-level carbapenem resistance but is now addressable through several newer BL/BLI combinations when the isolate remains susceptible (Tamma et al., 2024). OXA-48-like enzymes present additional diagnostic challenges because they may generate subtle or heterogeneous carbapenem minimum inhibitory concentration (MIC) patterns, potentially delaying recognition of carbapenemase production and appropriate treatment selection (Findlay et al., 2015; Nordmann and Poirel, 2019). Moreover, β-lactamase activity is often amplified by reduced permeability: porin loss or alteration lowers periplasmic β-lactam concentrations, allowing hydrolysis to dominate even when enzymatic activity alone would be borderline (Livermore, 1995; Nordmann and Poirel, 2019; Pages et al., 2008). Thus, the clinically relevant question is not simply whether a β-lactamase is present, but which enzyme is expressed, whether its catalytic mechanism falls within the inhibitor spectrum, and whether envelope changes have reduced drug exposure enough to convert a theoretically active BL/BLI combination into a compromised regimen.

This mechanism-resolved framework links β-lactamase biology directly to clinical failure modes. Delayed recognition of OXA-48-like or MBL-producing organisms can prolong ineffective empiric therapy; MBL production can restrict therapeutic options and preserve toxicity trade-offs even after the resistance mechanism is identified; and the combined β-lactamase production with porin loss can lead to apparent susceptibility–efficacy discordance or on-therapy resistance amplification. Table 1 summarizes the major β-lactamase categories, representative pathogens, and associated therapeutic logic, emphasizing that BL/BLI combinations should be selected through mechanism-resolved matching rather than indiscriminate escalation.

### Reduced permeability and porin alterations

Reduced outer-membrane permeability—mediated by porin loss, altered porin selectivity, or broader envelope remodeling—can dominate susceptibility outcomes by limiting periplasmic drug access, even when enzymatic resistance is modest (Davin-Regli et al., 2024; Delcour, 2009). In Enterobacterales, loss or remodeling of major porins such as OmpK35/OmpK36 can reduce uptake of β-lactams and other hydrophilic agents, whereas in non-fermenters such as *P. aeruginosa*, loss of the OprD pathway is particularly relevant to carbapenem resistance (Pages et al., 2008; Rocker et al., 2020; Wang et al., 2025). Permeability defects frequently synergize with β-lactamases: hydrolysis becomes more efficient when fewer drug molecules reach the periplasm, and inhibitor–enzyme advantages may be blunted when drug exposure at the site of action is insufficient (Hamzaoui et al., 2018; Krishnamoorthy et al., 2017; Livermore, 1995; Nordmann and Poirel, 2019; Pages et al., 2008). Clinically, permeability-driven resistance contributes to discordance between in vitro susceptibility expectations and therapeutic performance, especially when borderline MICs are interpreted without accounting for envelope context (Parsels et al., 2021; Tamma et al., 2024).

Therapeutic responses to permeability defects should be divided into three mechanistically distinct categories. First, barrier-bypassing or uptake-exploiting strategies use alternative entry routes rather than restoring native porin permeability. Cefiderocol is the clearest approved example; as a siderophore cephalosporin, it exploits bacterial iron transport systems to cross the outer membrane and can retain activity against some isolates in which porin loss or efflux reduces the activity of conventional β-lactams (Ito et al., 2018; Parsels et al., 2021; Zhanel et al., 2019). However, this approach does not restore permeability, because activity still depends on functional iron uptake pathways and can be affected by changes in siderophore receptor expression or other cefiderocol-specific resistance mechanisms (Ito et al., 2018; Karakonstantis et al., 2022). Second, exposure-augmenting strategies, including optimized dosing, prolonged infusion of time-dependent β-lactams, and selected combination regimens, may improve pharmacodynamic target attainment but do not restore outer-membrane permeability. Their effectiveness is therefore conditional on residual susceptibility, infection site, MIC, and host pharmacokinetics rather than true reversal of the permeability defect (Tamma et al., 2024). Third, permeability-restoring strategies remain largely experimental. Efflux pump inhibitors and outer-membrane permeabilizers can increase intracellular or periplasmic drug exposure in vitro, but no broadly applicable, clinically approved agent currently restores Gram-negative permeability in routine practice. Toxicity, specificity, pharmacokinetic limitations, and translational barriers remain major constraints (Lee et al., 2026; Li et al., 2015). Thus, when permeability is the dominant resistance driver, mechanism-informed therapy should distinguish true uptake bypass from exposure optimization and acknowledge that clinically validated permeability-restoring interventions remain an unmet need.

### Efflux-driven multidrug resistance

Upregulation of efflux pumps can confer broad, cross-class resistance by actively exporting structurally diverse antibiotics, thereby yielding multidrug-resistant phenotypes that are not reliably addressed by incremental class switching. In Gram-negative bacteria, RND family systems (e.g., AcrAB-TolC and MexAB-OprM) are particularly impactful because they couple inner membrane transport to outer membrane channels, enabling efficient clearance of drugs from both the periplasm and cytoplasm (Du et al., 2018; Mazza et al., 2024; Zgurskaya et al., 2018). Importantly, efflux acts synergistically with the outer membrane permeability barrier; therefore, modest increases in efflux can translate into disproportionately reduced intracellular exposure and clinically relevant non-susceptibility patterns (Krishnamoorthy et al., 2017; Zgurskaya et al., 2018). In addition to elevating MICs, high-efflux states can increase evolvability by promoting the accumulation of additional resistance mutations, which helps explain the fragility of monotherapy when residual activity is weakened by the active export (Bhattacharyya et al., 2022). Clinically, an efflux contribution is suggested by broad co-resistance profiles and can be compounded by concomitant permeability defects. Moreover, transmissible RND determinants such as TMexCD-TOprJ illustrate that efflux can be an acquired, spreading resistance mechanism (Dong et al., 2022). These considerations support mechanism-aware combination strategies for severe infections and motivate adjunctive approaches, including efflux pump inhibitors and nontraditional modalities, although translation has been limited by toxicity and pharmacokinetic challenges (Duffey et al., 2024; Mahey et al., 2024).

### Target modification and adaptive resistance

Target modification is a direct, high-impact route to resistance because it reduces drug binding at essential cellular targets, often leading to the failure of entire therapeutic classes. Fluoroquinolone resistance exemplifies this mechanism: mutations in the quinolone resistance-determining regions of gyrase and topoisomerase IV, together with plasmid-mediated quinolone resistance determinants, can weaken both intravenous efficacy and oral step-down pathways that depend on preserved target susceptibility (Hooper and Jacoby, 2015; Rodriguez-Martinez et al., 2016). Ribosome-targeting agents face analogous constraints. In Gram-negative pathogens, acquired rRNA methyltransferases are particularly consequential because they modify key rRNA nucleotides and can confer high-level resistance that compromises aminoglycosides as combination partners, thereby narrowing the options for severe infections in which synergistic regimens are often needed (Jeremia et al., 2023; Yang and Hu, 2022). Adaptive resistance adds an additional layer of therapeutic fragility in last-line settings, as polymyxin pressure can select for lipid A remodeling through chromosomal regulatory pathways (e.g., PhoPQ/PmrAB) while mobilized *mcr* genes provide an additional mechanism of resistance, jointly undermining polymyxin activity and intensifying toxicity–efficacy trade-offs (Huang et al., 2020; Hussein et al., 2021; Liu et al., 2016; Poirel et al., 2017). Because these target and envelope adaptations frequently coexist with β-lactamases, efflux, or permeability defects, “more of the same” strategies rarely restore activity once binding or target access is fundamentally compromised. Instead, these mechanisms motivate mechanism-informed class switching, combinations that reduce selective pressure, and nontraditional strategies that decouple clinical benefits from direct growth inhibition.

## Clinical Impact Framed as Mechanism-Defined Failure Modes

The clinical burden of AMR in Gram-negative infections is best understood not simply as a matter of high resistance rates, but as a predictable set of mechanism-defined failure modes that repeatedly constrain bedside decision-making. Resistance determinants reshape the early therapeutic window by increasing the likelihood of delayed effective therapy, a factor consistently associated with poor outcomes and increased resource utilization when appropriate coverage is not promptly achieved (Bassetti et al., 2022). As susceptibility becomes limited, clinicians are compelled to consider restricted treatment options that often carry substantial toxicity liabilities. Thus, the balance between therapeutic efficacy and potential harm has become a central feature in the management of modern multidrug-resistant Gram-negative bacterial infections (Paul et al., 2022; Tamma et al., 2024). Finally, even when in vitro activity appears favorable, persistent phenotypes—including biofilm-associated tolerance and persister-mediated survival—can drive recurrence and prolonged infection courses, highlighting the importance of source control and anti-biofilm principles for therapeutic success (Ciofu et al., 2022). Accordingly, this review organizes resistance and tolerance mechanisms into three clinically actionable failure modes: delayed effective therapy; restricted options and toxicity trade-offs; and persistence, biofilm, and recurrence.

### Delayed effective therapy

A central clinical consequence of mechanistically heterogeneous resistance in Gram-negative bacteria is delayed effective therapy, because empiric regimens may fail to match the pathogen’s dominant resistance architecture. These delays arise not simply from the presence of “resistance,” but from uncertainty about whether the dominant mechanism involves enzymatic hydrolysis, reduced permeability, efflux amplification, target modification, or mechanism stacking, each of which has distinct therapeutic implications. During the initial treatment window, empiric therapy must therefore remain risk-informed rather than purely mechanism-resolved, incorporating illness severity, infection syndrome, prior colonization or infection history, recent antibiotic exposure, healthcare exposure, and local epidemiology (Paul et al., 2022; Tamma et al., 2024).

The interventions to reduce delayed effective therapy operate at different levels and should not be conflated. At the patient level, empiric selection must be made before definitive susceptibility or mechanism data are available, especially in severe infections. At the diagnostic level, rapid genotypic assays can shorten the time to detection of major enzymatic determinants such as *bla*_KPC_, *bla*_NDM_, and *bla*_OXA-48-like_; however, they do not reliably capture porin loss, efflux upregulation, inducible regulatory responses, or the combined effects of multiple mechanisms in a single isolate (Banerjee and Patel, 2023; Hattab et al., 2024). Thus, rapid genotypic results may support early escalation or de-escalation when a determinant is identified, but they cannot fully replace phenotypic susceptibility testing or clinical interpretation of the envelope context. At the institutional level, antimicrobial stewardship programs help translate microbiological results into timely regimen modification and reduce unnecessary selection pressure, but they do not by themselves address the immediate bedside problem of empiric therapy selection for individual patients (Barlam et al., 2016; Peri et al., 2024). This distinction reframes mechanism-informed therapy as a staged process comprising initial risk-informed empiric coverage, rapid diagnostic-guided refinement, phenotypic confirmation, and stewardship-supported escalation or de-escalation as organism- and mechanism-level information becomes available (Banerjee et al., 2021; Peri et al., 2024; Pliakos et al., 2018).

### Restricted options and toxicity trade-offs

As resistance mechanisms accumulate, therapeutic options contract toward fewer agents that may be less effective, less tolerated, or more complex to dose (Paul et al., 2022; Tamma et al., 2024). When first-line β-lactams are compromised by β-lactamases, permeability defects, or efflux, treatment may shift toward last-line agents such as polymyxins, aminoglycosides, or complex combination regimens, thereby increasing nephrotoxicity, therapeutic drug monitoring requirements, and uncertainty in exposure–toxicity balance (Eljaaly et al., 2021; Paul et al., 2022; Tsuji et al., 2019). This toxicity trade-off is not merely a patient-level adverse-effect problem; it is a systems-level consequence of mechanism-driven resistance that pushes practice toward agents with narrower therapeutic indices or less predictable clinical performance (Paul et al., 2022; Tamma et al., 2024).

The benefit of mechanism-informed selection is therefore time-dependent. In the initial treatment window, empiric therapy must be started before organism identity, susceptibility profile, and resistance mechanism are fully resolved. Mechanism-resolved information becomes actionable only after microbiological data become available, including organism identification, phenotypic susceptibility testing, carbapenemase detection, or genotypic identification of key determinants (Bassetti et al., 2021; Hakeam et al., 2021; van Duin et al., 2018). Accordingly, clinical studies reporting improved outcomes or safety with newer targeted agents compared with colistin-based regimens should be interpreted as evidence for early definitive or microbiology-informed therapy, rather than as evidence that empiric uncertainty can be eliminated. For example, studies comparing ceftazidime–avibactam with colistin-based therapy in carbapenem-resistant Enterobacterales (CRE), and meropenem–vaborbactam with best available therapy in CRE, are most clinically informative when the CRE phenotype, KPC association, or susceptibility information has become available (Tamma et al., 2024; van Duin et al., 2018; Wunderink et al., 2018). Similarly, cefiderocol studies support its use as a targeted option for selected carbapenem-resistant Gram-negative infections; however, patient selection still depends on organism, susceptibility profile, infection site, and competing safety concerns (Bassetti et al., 2021; Tamma et al., 2024).

This limitation is especially important for MBL-producing organisms. Identification of an NDM-, VIM-, or IMP-producing isolate clarifies why standard BL/BLI combinations may fail, but does not automatically provide a low-toxicity solution because most approved BLIs do not directly inhibit MBLs (Boyd et al., 2020; Park et al., 2026). Aztreonam-avibactam-based strategies can partially address this gap by combining an MBL-stable monobactam with inhibition of co-produced SBLs; however, their clinical use still depends on availability, indication, susceptibility profile, species, infection site, and local access (Boyd et al., 2020; Tamma et al., 2024). Thus, mechanism-informed therapy can reduce unnecessary exposure to toxic or inactive agents once actionable data are available, but it cannot fully eliminate toxicity trade-offs in all resistance architectures. The practical value of mechanism resolution is therefore best framed as enabling timely transition from empiric coverage to active, mechanism-compatible definitive therapy, while acknowledging that some mechanisms—particularly MBL production and carbapenem-resistant *Acinetobacter baumannii* (CRAB)—remain major therapeutic gaps (Boyd et al., 2020; Tamma et al., 2024).

### Persistence, biofilm, and recurrence

Persistence and biofilm-associated tolerance are clinically important failure modes that are often underrecognized by standard antimicrobial susceptibility testing, which is typically performed on planktonic cells and may not predict the antibiotic exposure required to eradicate biofilm-embedded populations (Coenye, 2023; Donlan and Costerton, 2002). Mechanistically, persistence reflects phenotypic heterogeneity rather than stable resistance, and is often linked to transient growth arrest, reduced translation, ATP depletion, and metabolic downshifts that limit the activity of bactericidal antibiotics against their active targets (Lewis, 2010; Niu et al., 2024). Several stress-response pathways have been implicated in this state, including the stringent response mediated by (p)ppGpp, toxin-antitoxin systems, and SOS-associated dormancy programs. In *Escherichia coli*, the SOS-induced toxin TisB provides a well-characterized example, in which DNA damage signaling promotes membrane depolarization, collapse of the proton motive force, and ATP reduction, thereby driving multidrug-tolerant persister formation (Dorr et al., 2010; Podlesek and Zgur Bertok, 2020). In biofilm settings, these dormant subpopulations are further stabilized by matrix-associated diffusion limitation and by signaling programs such as cyclic di-GMP, which reinforce the transition toward a sessile, protected lifestyle (Ciofu et al., 2022; Flemming and Wingender, 2010; Stewart and Franklin, 2008). In device-associated and chronic airway infections, the extracellular matrix, diffusion limitation, altered metabolic states, and steep nutrient/oxygen gradients create microenvironments where effective drug penetration and activity are reduced, sustaining sublethal concentrations that favor tolerance and persister survival (Flemming and Wingender, 2010; Lewis, 2010; Stewart and Franklin, 2008). These biofilm phenotypes can also interact with classical resistance mechanisms—such as efflux and permeability barriers—further reducing local drug exposure and promoting the selection of adaptive responses within protected niches (Ciofu et al., 2022). Clinically, the consequence is not always immediate categorical non-susceptibility but failure to sterilize the infected site, leading to relapse after apparently “appropriate” therapy (Ciofu et al., 2022; Hall-Stoodley et al., 2004). Therefore, this failure mode supports strategies that pair antimicrobial choice with decisive source control and, where feasible, adjunctive approaches that disrupt biofilm structure or trigger dispersion to restore susceptibility (Donlan and Costerton, 2002; Rumbaugh and Sauer, 2020). Accordingly, the resistance–therapy matching framework summarized in Fig. 3 is introduced to translate mechanistic patterns of persistence and resistance into actionable therapeutic strategy classes.

## Therapeutic Strategies Aligned with Resistance Mechanisms

The clinical management of resistant Gram-negative infections increasingly depends on matching therapy to the dominant resistance architecture rather than escalating empirically in a class-blind manner. Contemporary guidelines emphasize mechanism-aware selection and timely de-escalation once susceptibility and resistance determinants are identified, reflecting both stewardship and outcome considerations (Paul et al., 2022; Tamma et al., 2024). Within this framework, therapeutic strategies can be categorized as follows: (i) mechanism-matched BL/BLI regimens that exploit predictable inhibitor–enzyme relationships; (ii) barrier-bypassing or uptake-exploiting agents designed to retain activity despite permeability constraints; (iii) nontraditional modalities such as phages, antivirulence, or microbiome-based approaches that aim to circumvent classic resistance pathways; and (iv) cross-cutting tactics, including combination therapy and pharmacokinetics/pharmacodynamics (PK/PD) optimization, that widen exposure margins and reduce the risk of on-therapy failure. Recent guideline updates also highlight persistent evidence gaps—particularly for new agents used in high-severity infections—making transparent reasoning about the mechanism and exposure central to rational therapeutic decision making (see Fig. 2, Table 1) (Paul et al., 2022). To clarify the logic in Fig. 3, the effectiveness indicators are intended as conceptual and relative assessments of how directly each strategy addresses the dominant resistance mechanism; the rationale for individual assignments is summarized in Table S1.

### Mechanism-matched BL/BLI therapy

Newer BL/BLI combinations exemplify the transition from broad escalation to mechanism-informed therapy, but their clinical value depends on more than matching an inhibitor to an enzyme class (Bush and Bradford, 2019; Tooke et al., 2019). Current European Society of Clinical Microbiology and Infectious Diseases (ESCMID) and Infectious Diseases Society of America (IDSA) guidance already incorporates mechanism-aware recommendations for resistant Gram-negative infections. Therefore, the key question for this review is not whether BL/BLI therapy should be mechanism matched, but where this approach fails, becomes unstable, or requires additional clinical information (Paul et al., 2022; Tamma et al., 2024). Structural and catalytic aspects of β-lactamases and β-lactamase inhibitors have recently been reviewed in detail; therefore, the present section focuses on the clinical gaps that determine whether an apparently rational BL/BLI choice remains effective in practice (Park et al., 2026).

First, mechanism matching is dynamic because resistance can emerge during therapy. Ceftazidime–avibactam is a rational choice for many KPC-producing Enterobacterales when susceptibility is retained, but treatment-emergent resistance can occur through mutations in *bla*_KPC_ that alter the KPC enzyme and reduce avibactam-mediated protection (Shields et al., 2017; Tamma et al., 2024). This example illustrates that an initial inhibitor–enzyme match should not be considered a fixed solution. Persistent bacteremia, microbiological relapse, rising MICs, or clinical non-response should prompt repeat culture, susceptibility reassessment, and reconsideration of the resistance architecture, including possible enzyme evolution, porin loss, efflux, or mixed populations (Pereira et al., 2021; Shields et al., 2017; Tamma et al., 2024).

Second, BL/BLI selection is pathogen- and site-specific. In Enterobacterales, the distinction among KPC, OXA-48-like, and MBL mechanisms remains central because each predicts a different inhibitor spectrum and therapeutic pathway. In *P. aeruginosa*, ceftolozane–tazobactam has a distinct role because ceftolozane retains intrinsic activity against many resistant *P. aeruginosa* isolates, whereas ceftazidime–avibactam may be favored in selected contexts depending on susceptibility, local epidemiology, and prior exposure (Tamma et al., 2024). For hospital-acquired and ventilator-associated pneumonia, infection-site pharmacology must also be considered because lung epithelial lining fluid penetration, dosing intensity, renal clearance, MIC, and illness severity can determine whether an in vitro active BL/BLI achieves sufficient exposure at the infection site (Caro et al., 2020; Tamma et al., 2024). In contrast, CRAB remains poorly addressed by most conventional BL/BLI combinations; sulbactam-based activity is pathogen-specific, and recent sulbactam–durlobactam data highlight that CRAB requires a distinct therapeutic logic rather than simple extrapolation from CRE or *P. aeruginosa* treatment pathways (Kaye et al., 2023; Tamma et al., 2024).

Third, the role of carbapenems within a mechanism-informed framework should be explicit. Some BL/BLI combinations are best understood as carbapenem-restoring strategies. Meropenem–vaborbactam and imipenem–relebactam restore carbapenem activity primarily against selected serine carbapenemase-producing or imipenem-nonsusceptible organisms, particularly when the relevant β-lactamase is within the inhibitor spectrum and susceptibility is retained (Motsch et al., 2020; Wunderink et al., 2018). By contrast, carbapenem-sparing strategies may be preferable when a non-carbapenem BL/BLI is active, when stewardship aims to reduce carbapenem exposure, or when the resistance mechanism predicts poor carbapenem restoration, such as MBL production. However, this distinction is not absolute; severity of illness, infection site, source control, MIC, prior therapy, renal function, and local susceptibility patterns may justify carbapenem-containing or carbapenem-sparing regimens in different patients (Paul et al., 2022; Tamma et al., 2024).

Finally, PK/PD optimization should be viewed as complementary to mechanism matching rather than a substitute for it. Prolonged or optimized infusion of time-dependent β-lactams can improve pharmacodynamic target attainment when susceptibility is retained or MICs approach the susceptibility breakpoint, but it cannot overcome a fundamentally inactive inhibitor–enzyme pairing or absent drug entry (Abdul-Aziz et al., 2024; Dulhunty et al., 2024; Roberts et al., 2014). Thus, BL/BLI therapy is best framed as an iterative process: identify the most plausible resistance mechanism, select an inhibitor–backbone pair with mechanistic plausibility and demonstrated susceptibility, account for pathogen and infection site, optimize exposure, and reassess when clinical or microbiological response is inadequate (Roberts et al., 2014; Tamma et al., 2024).

### Barrier-bypassing antibiotics and uptake exploitation

When reduced permeability or efflux dominates susceptibility, therapeutic success may depend on strategies that bypass classical envelope barriers rather than intensifying conventional β-lactam exposure (Delcour, 2009; Krishnamoorthy et al., 2017). Barrier-bypassing concepts include exploiting alternative uptake pathways and selecting agents whose entry is less dependent on specific porins or whose activity is less susceptible to efflux, thereby reframing “poor penetration” as a solvable, mechanism-defined constraint (Davin-Regli et al., 2024; Krishnamoorthy et al., 2017). A practical example of uptake exploitation is siderophore-mediated transport, in which drug entry is coupled to bacterial iron acquisition systems (a “Trojan horse” mechanism) and can partially decouple activity from baseline outer membrane permeability (Parsels et al., 2021; Sato and Yamawaki, 2019). This framework helps explain why performance differs across Enterobacterales and non-fermenters with distinct envelope and uptake architectures and further clarifies why clinical efficacy may be infection-site and pathogen-group dependent rather than being uniformly applicable across “Gram-negative bacteria” (Bassetti et al., 2021; Wunderink et al., 2021). Importantly, barrier bypass is not universal; its clinical value is highest when resistance is exposure-limited (permeability/efflux-dominant) rather than target-limited and when relevant uptake pathways remain functional—an assumption that can fail as resistance emerges via changes in iron transport and related entry dominance (Bianco et al., 2024; Kocer et al., 2024). In this review, barrier-bypassing agents are treated as a distinct strategy class within the mechanism map. This classification is explicitly linked to the permeability and efflux modules discussed above and helps guide rational combination design when multi-mechanism resistance reduces the robustness of any single agent (Parsels et al., 2021; Tamma et al., 2024).

### Nontraditional therapeutics: Phages, antivirulence, and microbiome-based interventions

Nontraditional modalities such as therapeutic bacteriophages, antivirulence, and microbiome-based interventions provide mechanistically distinct options that can complement antibiotics when classical resistance determinants restrict choices or when biofilm/persistence phenotypes dominate. Phages enable targeted bacterial killing with pharmacological properties shaped by host range, in situ amplification, and, in some preparations, biofilm-depolymerase activity; however, clinical outcomes remain heterogeneous across indications and trial designs, underscoring the need for better-standardized products and fit-for-purpose endpoints (Jault et al., 2019; Sawa et al., 2024; Uyttebroek et al., 2022). In contrast, antivirulence strategies aim to disarm pathogenic functions, including quorum sensing, secretion systems, adhesion, and biofilm programs, potentially shifting the therapeutic goal from growth inhibition to fitness reduction and improved host clearance; this conceptual advantage should be interpreted cautiously because efficacy is context-dependent and the evidentiary base remains dominated by preclinical and early translational studies (Fleitas Martinez et al., 2019; Liao et al., 2022; Naga et al., 2023; Ogawara, 2021). Microbiome-based interventions also merit explicit consideration within this framework because the intestinal microbiota serves as an ecological barrier against colonization by multidrug-resistant Gram-negative organisms, and disruption of this barrier can precede endogenous infection and onward transmission. Rather than replacing active systemic therapy for established invasive infection, these approaches aim to restore colonization resistance through ecological competition, metabolic exclusion, and host-microbiota interactions (Caballero-Flores et al., 2023; Davido et al., 2025).

The clinical maturity of these nontraditional modalities remains uneven. Among phage-based approaches, the most visible controlled clinical experience is still the PhagoBurn trial, a randomized phase I/II study in *P. aeruginosa* burn-wound infection, which demonstrated feasibility and acceptable tolerability but also highlighted substantial manufacturing and potency-standardization challenges; subsequent systematic reviews indicate growing compassionate-use and observational experience, but no broadly adopted, standardized phage product for multidrug-resistant Gram-negative infection is yet close to routine market use (Jault et al., 2019; Sawa et al., 2024; Uyttebroek et al., 2022). Antivirulence strategies are similarly heterogeneous in readiness. The most advanced programs are anti-*P. aeruginosa* biologics targeting PcrV-based virulence mechanisms: KB001-A underwent early clinical evaluation, MEDI3902 (gremubamab) completed phase I testing and a proof-of-concept randomized study in colonized mechanically ventilated patients, and the related bispecific antibody AZD0292 is currently in phase IIb testing in bronchiectasis with chronic *P. aeruginosa* colonization (Ali et al., 2019; Chastre et al., 2022; ClinicalTrials.gov., 2025; Jain et al., 2018). By contrast, most quorum sensing and biofilm-directed small-molecule antivirulence programs remain preclinical, indicating that the field has not yet translated into near-market therapeutics outside selected biologic platforms. Microbiome-based interventions may be closest to practical deployment in terms of platform availability, because fecal microbiota transplantation and related microbiota-restoration approaches are already clinically implemented in other indications and are now being tested for multidrug-resistant organism decolonization; however, for resistant Gram-negative carriage specifically, current evidence is still limited to prospective cohorts, comparative studies, and early implementation trials rather than approved indication-specific products (Bar-Yoseph et al., 2021; Woodworth et al., 2025).

It is therefore crucial to identify the most plausible clinical niches for these approaches, including highly resistant infections with limited antibacterial alternatives, biofilm-associated diseases requiring adjunctive debulking, intestinal decolonization or prevention of recurrent infection, and scenarios in which reducing virulence or restoring colonization resistance may reduce the selection pressure and transmission risk. Finally, practical constraints—such as matching, manufacturing, delivery, donor or product standardization, engraftment durability, and regulatory oversight—should be explicitly acknowledged because they limit the scalability of real-world implementation even when the mechanistic rationale is strong (Davido et al., 2025; Fuerst-Wilmes et al., 2025; Sawa et al., 2024).

### Combination therapy and PK/PD optimization

Combination therapy and PK/PD optimization are often presented as cross-cutting strategies, but their roles differ according to pathogen, resistance architecture, infection site, and the availability of reliably active monotherapy (Paul et al., 2022; Tamma et al., 2024). Combination therapy should therefore be considered in at least three distinct clinical uses. First, it may be used for empiric broadening when the pathogen and resistance mechanism are not yet defined. Second, it may function as a targeted primary strategy in limited-option infections, particularly CRAB, where conventional monotherapy is often unreliable and sulbactam-based or sulbactam–durlobactam-containing regimens have a distinct pathogen-specific rationale (Kaye et al., 2023; Tamma et al., 2024). Third, combinations may be used to improve regimen robustness when heterogeneity, high bacterial burden, or mixed susceptibility patterns are suspected (Paul et al., 2022; Tamma et al., 2024). However, the ability of combination therapy to suppress resistance emergence remains unresolved and should not be assumed. Randomized data on older colistin–carbapenem combinations did not consistently demonstrate superior outcomes, while evolutionary studies indicate that drug combinations can influence resistance trajectories in complex and context-dependent ways (Bognar et al., 2024; Paul et al., 2018). Thus, combinations are most defensible when they address a defined therapeutic gap—such as inadequate activity of any single agent, pathogen-specific limited options, high-inoculum infection, or empiric uncertainty—rather than when used reflexively to “prevent resistance” (Paul et al., 2022; Tamma et al., 2024).

PK/PD optimization should be framed with similar caution. Prolonged or continuous infusion of time-dependent β-lactams can increase the probability of maintaining free drug concentrations above the MIC, and is particularly relevant in critically ill patients with altered volume of distribution, augmented renal clearance, renal replacement therapy, high MICs near the susceptible breakpoint, or deep-seated infections where exposure is uncertain (Roberts et al., 2014; Tamma et al., 2024). However, the clinical evidence is not fully concordant. A 2024 systematic review and Bayesian meta-analysis reported that prolonged β-lactam infusion was associated with reduced 90-day mortality in critically ill adults with sepsis or septic shock, whereas the BLING III randomized trial did not show a statistically significant 90-day mortality benefit for continuous versus intermittent β-lactam infusion in critically ill patients with sepsis (Abdul-Aziz et al., 2024; Dulhunty et al., 2024). This discrepancy supports a targeted rather than universal interpretation. PK/PD optimization is most compelling when susceptibility is retained and the expected problem is insufficient exposure, not when the drug–mechanism pairing is inactive, inhibitor spectrum is mismatched, or outer-membrane entry is absent (Roberts et al., 2014; Tamma et al., 2024).

Accordingly, combination therapy and PK/PD optimization should be integrated into mechanism-informed treatment as conditional tools rather than generic rules. For CRAB and other limited-option infections, combination therapy may serve as a primary strategy because the main problem is lack of a reliably active single agent (Kaye et al., 2023; Tamma et al., 2024). For susceptible but exposure-challenged infections, optimized infusion may help close pharmacodynamic gaps (Abdul-Aziz et al., 2024; Roberts et al., 2014). In contrast, neither approach should be presented as reliably reducing selection pressure in the absence of mechanism-specific evidence (Bognar et al., 2024; Paul et al., 2018). The practical sequence is therefore to define the dominant barrier to activity, select an active backbone when available, use combination therapy only when it addresses a specific coverage or robustness gap, and apply PK/PD optimization when the expected limitation is exposure rather than intrinsic inactivity (Paul et al., 2022; Tamma et al., 2024).

Platform-level development of antibiotic hybrids and adjuvant-based strategies has recently been reviewed elsewhere; therefore, the present section focuses on how combination therapy and PK/PD optimization should be interpreted within clinical resistance–therapy matching rather than on cataloguing individual drug-development platforms (Lee et al., 2026).

## Enabling Mechanism-Informed Therapy and Future Priorities

Mechanism-informed therapy is feasible only when diagnostic workflows generate information that is timely, interpretable, and actionable within the clinical decision window. In practice, this requires a staged approach rather than a fully mechanism-resolved initial decision. Empiric therapy must first be guided by severity of illness, infection syndrome, prior microbiology, recent antimicrobial exposure, healthcare exposure, and local epidemiology; mechanism-resolved refinement can then occur as organism identification, phenotypic susceptibility testing, and targeted resistance-determinant data become available (Paul et al., 2022; Tamma et al., 2024). Rapid genotypic assays can shorten the time to detection of selected enzymatic determinants, including major carbapenemase genes, but they do not reliably resolve porin loss, efflux upregulation, inducible regulatory responses, quantitative expression effects, or multi-mechanism stacking (Banerjee and Patel, 2023; Hattab et al., 2024; Pliakos et al., 2018). Therefore, rapid molecular results should be interpreted as accelerators of early refinement rather than complete replacements for phenotypic susceptibility testing or expert interpretation of the envelope and clinical context.

The implementation value of diagnostics also depends on whether results are connected to timely therapeutic action. Clinical and economic analyses of bloodstream infection diagnostics suggest that rapid diagnostic testing is most useful when linked to antimicrobial stewardship programs that support interpretation, escalation, de-escalation, and avoidance of redundant coverage (Peri et al., 2024; Pliakos et al., 2018). Stewardship should therefore be framed not as a bedside substitute for empiric decision-making, but as an institutional mechanism for translating microbiological information into safer and more selective prescribing. This distinction is central to the framework proposed here, mechanism-informed therapy begins with risk-informed empiric coverage, proceeds through diagnostic-guided refinement and phenotypic confirmation, and ends with stewardship-supported selection-pressure management.

Several diagnostic and therapeutic gaps remain priority targets for future research. First, diagnostics must move beyond binary detection of selected resistance genes toward integrated genotype–phenotype interpretation that can account for β-lactamase class, porin loss, efflux, target modification, heteroresistance, and mechanism stacking. Second, therapeutic evidence must become more pathogen- and site-specific, particularly for MBL-producing Enterobacterales, difficult-to-treat resistant *P. aeruginosa*, CRAB, and infections in compartments where drug exposure is uncertain, such as pneumonia or deep-seated infections (Tamma et al., 2024; WHO, 2024). Third, future trials should evaluate not only clinical cure or mortality but also microbiological durability, on-therapy resistance emergence, toxicity trade-offs, and whether diagnostic-guided strategies can shorten ineffective therapy without encouraging unnecessary broad-spectrum exposure. These priorities align with global pathogen-ranking exercises that identify CRAB, resistant Enterobacterales, and other difficult-to-treat Gram-negative pathogens as continuing public health priorities (WHO, 2024).

Synthetic biology-assisted diagnostic and antimicrobial technologies including CRISPR-Cas-based approaches and engineered bacteriophage or microbiome strategies, have recently been reviewed as emerging platforms (Oh et al., 2026). In the present review, however, these approaches are considered primarily in relation to the current implementation gap because most routine workflows still depend on a staged combination of empiric risk assessment, rapid detection of selected resistance determinants, phenotypic susceptibility testing, and stewardship-guided interpretation. Their near-term value will therefore depend on whether they can be integrated into this workflow, produce interpretable results within clinically meaningful timeframes, and demonstrate patient-level benefit beyond technical proof of concept.

In summary, Gram-negative AMR should be approached as a mechanistic system that generates predictable failure modes rather than as a static susceptibility label. Mechanism-resolved diagnosis and therapy matching can help convert empiric uncertainty into a staged clinical strategy—supporting targeted escalation and de-escalation, minimizing avoidable toxicity and selection pressure, and identifying the diagnostic and therapeutic gaps most likely to shape future AMR burden.

## Figures and Tables

**Fig. 1.**
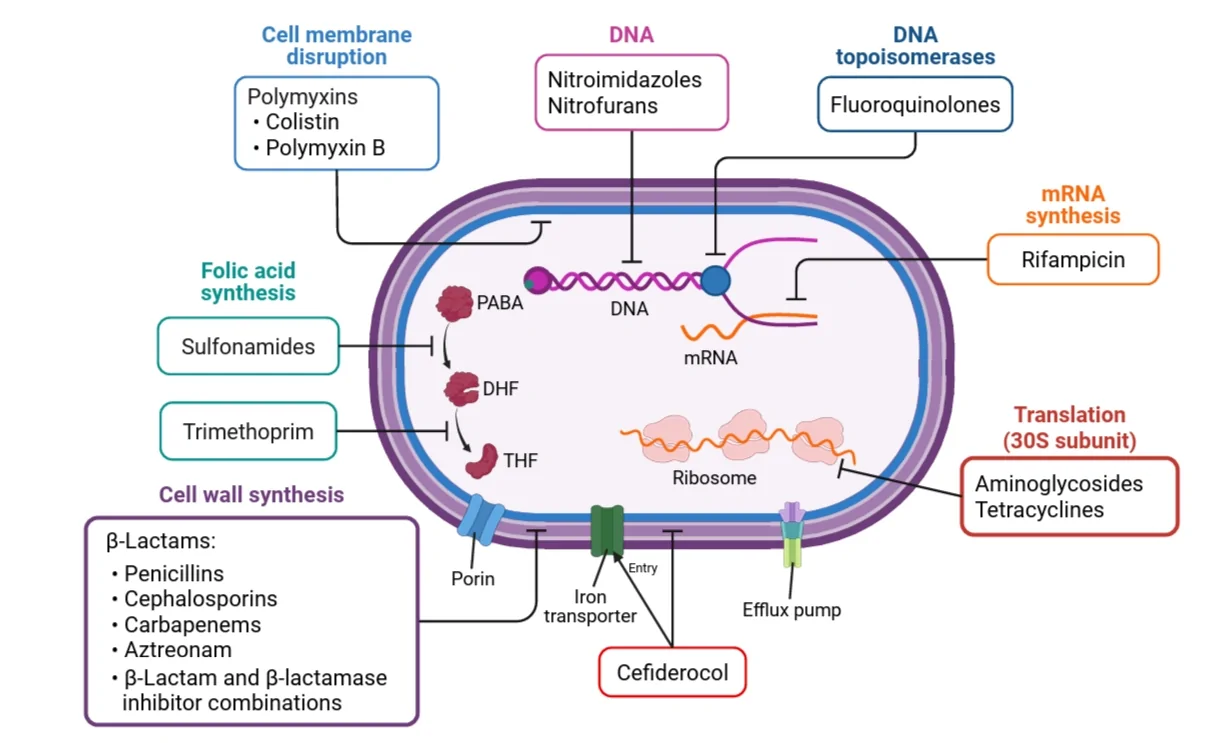
Mechanisms of action of antibacterial agents relevant to multidrug-resistant Gram-negative bacterial infections. Antibacterial classes target essential bacterial functions, including cell wall synthesis (β-lactams), cell membrane disruption (polymyxins), protein synthesis (aminoglycosides and tetracyclines), folate biosynthesis (sulfonamides and trimethoprim), and nucleic acid synthesis (fluoroquinolones targeting DNA topoisomerases; rifampicin inhibiting mRNA synthesis). Additional DNA-targeting agents shown in the figure include nitroimidazoles and nitrofurans, which require intracellular reduction for activation and induce DNA damage. The figure also highlights Gram-negative envelope features that shape drug exposure and therapeutic performance, including porins and efflux pumps, and illustrates an uptake-exploitation strategy using cefiderocol, which enters the cell via iron-transport pathways. β-Lactamase inhibitors are shown as adjuvants used in combination with β-lactams to restore activity against β-lactamase-producing pathogens. This figure was created using BioRender.com. PABA, para-aminobenzoic acid; DHF, dihydrofolic acid; THF, tetrahydrofolate.

**Fig. 2.**
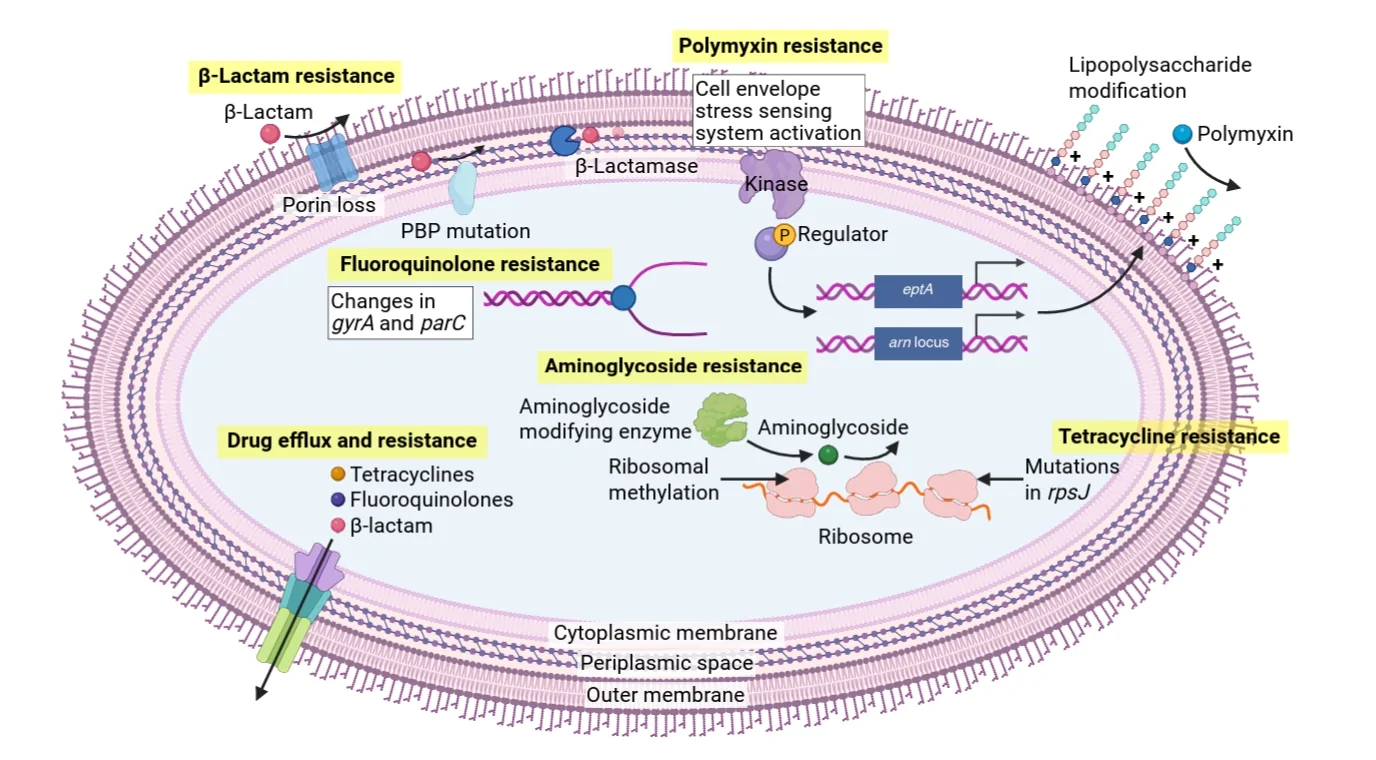
Key resistance mechanism modules in Gram-negative pathogens. The outer membrane of Gram-negative bacteria acts as a permeability barrier that restricts antibiotic access to targets in the periplasm and cytoplasm. The loss or remodeling of outer membrane porins reduces drug uptake and contributes to decreased susceptibility, particularly to β-Lactams. β-lactam resistance is further driven by β-lactamases (including carbapenemases) that hydrolyze the β-lactam ring, as well as target-site alterations such as penicillin-binding protein mutations that reduce β-lactam affinity. Activation of envelope stress-sensing systems can promote polymyxin resistance by inducing lipopolysaccharide modification, including pathways mediated by *eptA* and the *arn* locus. Aminoglycoside resistance arises through aminoglycoside-modifying enzymes and ribosome-associated mechanisms such as ribosomal methylation. Additional target-based resistance includes fluoroquinolone resistance via mutations in *gyrA* and *parC* and tetracycline resistance linked to mutations in *rpsJ*. Finally, multidrug efflux systems reduce intracellular drug exposure and contribute to the broad resistance pattern across multiple antibiotic classes, as shown in the figure. This figure was created using BioRender.com.

**Fig. 3.**
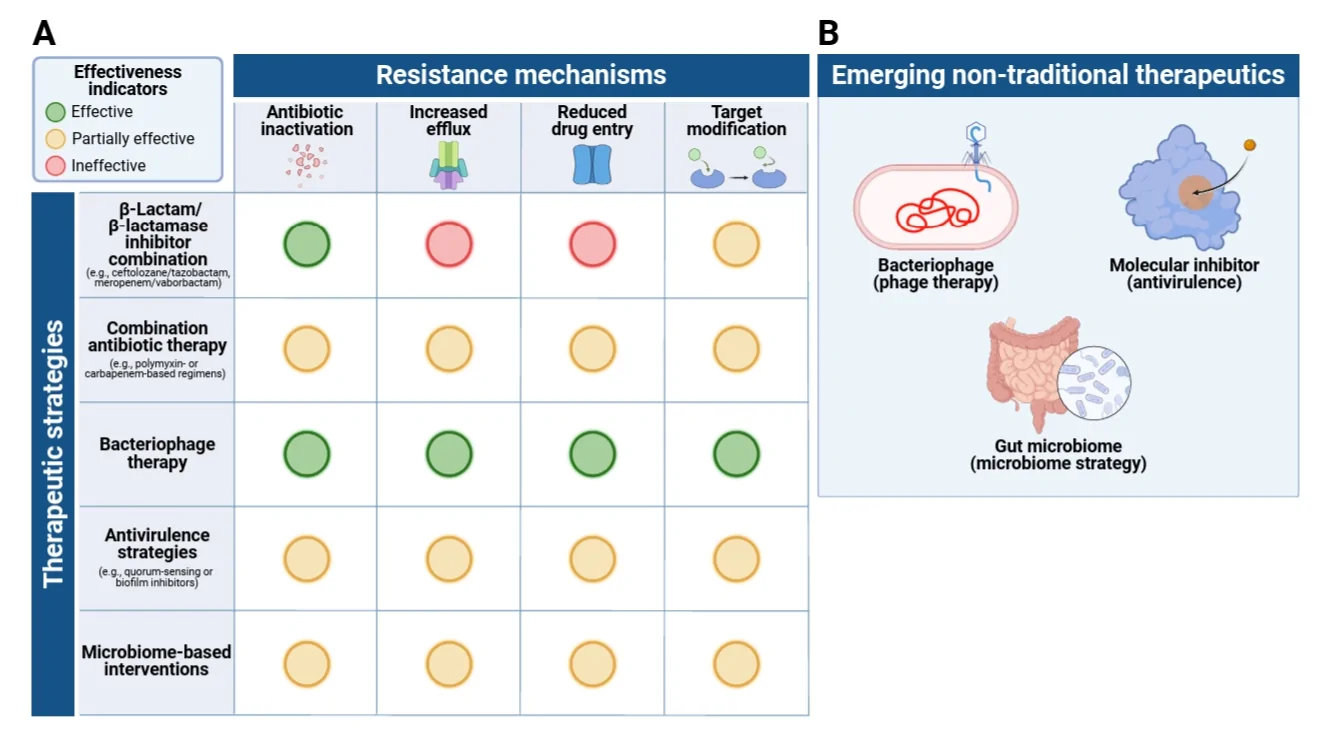
Mechanism-informed therapeutic strategies targeting antimicrobial-resistant Gram-negative bacteria. (A) Resistance-therapy matching map linking major Gram-negative resistance mechanisms—antibiotic inactivation, reduced drug entry (permeability/porin changes), increased efflux, and target modification—to therapeutic strategy classes. Strategies include BL/BLI combinations, combination antibiotic therapy, bacteriophage therapy, antivirulence strategies, and microbiome-based interventions. Effectiveness indicators denote conceptual and relative expected impact when the indexed mechanism is the dominant driver of failure: effective, direct neutralization, bypass, or mechanistic orthogonality; partially effective, context-dependent or adjunctive benefit without direct reversal of the mechanism; and ineffective, no consistent direct benefit expected when that mechanism predominates. In this framework, BL/BLI combinations are most effective against antibiotic inactivation, bacteriophage therapy is largely orthogonal to the classical antibiotic-resistance modules shown here, and combination regimens, antivirulence approaches, and microbiome-based interventions are interpreted primarily as adjunctive or context-dependent strategies. The rationale for individual assignments is summarized in Table S1. These indicators are intended to support hypothesis-driven selection and do not replace organism- and site-specific susceptibility testing, clinical severity assessment, or pharmacokinetic/pharmacodynamic optimization. (B) Representative examples of emerging nontraditional modalities are highlighted in this framework, including bacteriophages, molecular antivirulence inhibitors, and microbiome-based strategies. This figure was created using BioRender.com.

**Table 1.** Key resistance mechanisms in Gram-negative bacteria: clinical impact and therapeutic implications

Resistance mechanism (primary barrier)	Representative determinants (genes/systems)	Representative pathogens	Typical affected drug classes/phenotype^a^	Clinical impacts	Mechanism-informed therapeutic approach (strategy, examples)	References
β-Lactam hydrolysis: ESBL	*bla*_CTX-M _(± *bla*_TEM_/*bla*_SHV_)	*Escherichia coli*, *Klebsiella pneumoniae*	Third-generation cephalosporins, aztreonam (variable)	Empiric cephalosporin failure; step-up to broader agents	Carbapenem-sparing when appropriate; consider BL/BLI when supported by guidelines and susceptibility; avoid blind escalation	Bush and Bradford (2019); Husna et al. (2023); Tamma et al. (2024)
β-Lactam hydrolysis: AmpC	Chromosomal or plasmid AmpC (e.g., *bla*_CMY_), induction/derepression	*Enterobacter cloacae* complex, *Citrobacter freundii*, *Serratia marcescens*	Many cephalosporins; inducible resistance/inoculum effect	On-therapy resistance emergence; relapse risk	Avoid strong inducers and unstable cephalosporins; prefer definitive therapy guided by mechanism and AST	Cheo et al. (2025); Tamma et al. (2024); Tebano et al. (2024)
Carbapenemases: KPC (Class A)	*bla*_KPC_	*Klebsiella pneumoniae* (CRE), other Enterobacterales	Carbapenems; broad β-lactam resistance	Limited options; healthcare outbreaks	Use KPC-active BL/BLI as backbone when susceptible; de-escalate by AST; reinforce infection control	Di Bella et al. (2021); Li et al. (2021); Tamma et al. (2024)
Carbapenemases: MBL (Class B)	*bla*_NDM_/*bla*_VIM_/*bla*_IMP_	Enterobacterales, *Pseudomonas aeruginosa*	Carbapenems; most BL/BLI ineffective	High-level resistance; frequent therapeutic gaps	MBL-oriented strategies (e.g., aztreonam-based approaches when appropriate); consider combination in severe infections	Bassetti et al. (2020); Sangiorgio et al. (2025); Tamma et al. (2024)
Carbapenemases: OXA-type (Class D)	*bla*_OXA-48_-like (Enterobacterales), *bla*_OXA-23/24/58_ (*Acinetobacter baumannii*)	*Klebsiella pneumoniae* (OXA-48-like), *Acinetobacter baumannii*	Variable carbapenem resistance; subtle phenotypes	Missed detection; delayed active therapy	Mechanism-aware diagnostics; select active agents by AST; avoid carbapenem reliance at borderline MICs	Bonnin et al. (2025); Hirvonen et al. (2021); Tamma et al. (2024)
Reduced permeability (porin loss/alteration)	OmpK35/36 loss, OprD loss	*Klebsiella pneumoniae*, *Pseudomonas aeruginosa*	Carbapenems (e.g., imipenem with OprD loss); multiple β-lactams	Synergy with β-lactamases → high-level resistance	Prefer agents less dependent on specific porins; optimize PK/PD; consider combinations in severe disease	David et al. (2022); Rocker et al. (2020); Tamma et al. (2024)
Efflux pump upregulation (RND pumps)	AcrAB-TolC, MexAB-OprM, MexXY	*Escherichia coli*, *Pseudomonas aeruginosa*, *Acinetobacter baumannii*	MDR across classes (FQs, tetracyclines, some β-lactams, etc.)	Broad MDR; selection under therapy	Avoid fragile monotherapy when efflux-driven MDR suspected; exposure optimization; adjunct concepts (developmental)	Dulanto Chiang and Dekker (2024); Shi et al. (2025); Tamma et al. (2024)
Target modification: FQs	*gyrA*/*parC* mutations; *qnr* (± *aac(6’)*-*Ib*-*cr*)	Enterobacterales, *Pseudomonas aeruginosa*	FQ non-susceptibility	Loss of oral step-down options	Use nontarget-compromised classes; careful oral step-down selection; stewardship to reduce selection pressure	Hooper and Jacoby (2015); Kherroubi et al. (2024); Rodriguez-Martinez et al. (2016)
Ribosomal modification/protection (selected classes)	16S rRNA methylases (ArmA/Rmt), *tet* determinants (context-dependent)	*Acinetobacter baumannii*, Enterobacterales	Aminoglycosides (methylases); tetracyclines (variable)	Loss of combination partners; toxicity pressure	Avoid ineffective aminoglycosides “add-on”; choose alternatives with proven activity; toxicity-aware regimens	Tamma et al. (2024); Wagenlehner et al. (2019); Yang and Hu (2022)
Lipid A modification: polymyxin resistance	*mcr*; PhoPQ/PmrAB alterations	Enterobacterales, *Acinetobacter baumannii*	Polymyxins/colistin	Compromises last-line therapy	Restrict polymyxins; prioritize alternative active agents and source control; combinations only when evidence supports	Liu et al. (2016); Tamma et al. (2024); WHO (2024)
Biofilm-associated tolerance/persistence	c-di-GMP networks; EPS matrix; persister formation (multifactorial)	*Pseudomonas aeruginosa*, device-associated Enterobacterales	Phenotypic tolerance (MIC may not predict)	Chronic/relapsing infection	Source control (removal/drainage) + active agents; tailor duration to syndrome/site	Grooters et al. (2024); Tamma et al. (2024); Zafer et al. (2024)
Horizontal gene transfer & clonal spread	Plasmids; integrons; transposons; high-risk clones	CRE, CRPA, CRAB	Rapid cross-species dissemination	Outbreak propagation; repeated introductions	Surveillance + infection prevention; stewardship framed by selection pressure/transmission	Bhat et al. (2023); Wang et al. (2024); WHO (2024)

Examples are representative and not exhaustive. ^a^Affected drug classes/phenotype reflects typical patterns and may vary by species, co-mechanisms (e.g., porin loss plus β-lactamase), and local breakpoints. ESBL, extended-spectrum β-lactamase; BL, β-lactam; BLI, β-lactamase inhibitor; AST, antimicrobial susceptibility testing; CRE, carbapenem-resistant Enterobacterales; MBL, metallo-β-lactamase; OXA, oxacillinase; MIC, minimum inhibitory concentration; PK/PD, pharmacokinetics/pharmacodynamics; FQ, fluoroquinolone; CRPA, carbapenem-resistant *Pseudomonas aeruginosa*; CRAB, carbapenem-resistant *Acinetobacter baumannii*; KPC, *Klebsiella pneumoniae* carbapenemase; MDR, multidrug resistance; EPS, extracellular polymeric substances; RND, resistance-nodulation-division.

## Supplementary Information

Table S1. Rationale for the effectiveness assignments used in Fig. 3A. This table summarizes the conceptual basis for the effectiveness indicators used in Fig. 3A. Assignments were made according to whether a therapeutic strategy directly neutralizes or bypasses the indexed resistance mechanism, provides context-dependent or adjunctive benefit without directly reversing the mechanism, or is not expected to confer a consistent direct advantage when that mechanism is dominant. The table is intended to support interpretation of the resistance-therapy matching framework and should not be read as a prescriptive clinical algorithm.
