# Supplementary material for: From resistance mechanisms to therapy: Antimicrobial resistance in Gram-negative bacteria
Source: J Microbiol. 2026 Aug 6;64(8):e2604017. doi: 10.71150/jm.2604017 (PMC13688131; doi:10.71150/jm.2604017)
Supplement: Supplementary file 1 [file jm-2604017-Supplementary-Table-S1.pdf]

Table S1. Rationale for the effectiveness assignments used in Fig. 3A. This table summarizes the conceptual basis for the effectiveness indicators used in Fig. 3A. Assignments were made according to whether a therapeutic strategy directly neutralizes or bypasses the indexed resistance mechanism, provides context-dependent or adjunctive benefit without directly reversing the mechanism, or is not expected to confer a consistent direct advantage when that mechanism is dominant. The table is intended to support interpretation of the resistance-therapy matching framework and should not be read as a prescriptive clinical algorithm.

Therapeutic strategy	Resistance mechanism				Brief rationale	References
	Antibiotic inactivation	Reduced drug entry	Increased efflux	Target modification		
**β-Lactam/β-lactamase inhibitor combinations**	Effective[a]	Ineffective[c]	Ineffective	Partially effective[b]	Directly addresses inhibitor-susceptible β-lactamase-mediated hydrolysis, especially serine β-lactamases, and therefore represents the clearest mechanism-matched response when antibiotic inactivation is dominant. Does not restore porin-mediated uptake or reverse active export, and only limited indirect benefit is expected when altered target affinity contributes to failure.	Bush and Bradford (2019); Krishnamoorthy et al. (2017); Tamma et al. (2024)
**Combination antibiotic therapy**	Partially effective	Partially effective	Partially effective	Partially effective	Combination regimens may improve robustness, broaden coverage, or reduce on-therapy failure, but they usually do not directly reverse the indexed resistance mechanism. Benefit is therefore context-dependent and regimen-specific rather than universally mechanism-corrective.	Krishnamoorthy et al. (2017); Paul et al. (2022); Tamma et al. (2024)
**Bacteriophage therapy**	Effective	Effective	Effective	Effective	Phage activity is mechanistically orthogonal to canonical antibiotic resistance modules. β-lactamase production, efflux, porin loss, and antibiotic target mutations do not directly impair phage absorption, replication, or lytic killing, although	Sawa et al. (2024); Uyttebroek et al. (2022)

					separate phage-resistance may still emerge.	
**Antivirulence strategies**	Partially effective	Partially effective	Partially effective	Partially effective	Antivirulence strategies generally reduce pathogenic fitness, tissue damage, biofilm resilience, or host-pathogen interaction rather than directly reversing canonical resistance determinants. Their value is therefore mainly adjunctive and context-dependent across mechanism classes.	Fleitas Martinez et al. (2019); Liao et al. (2022); Ogawara, (2021)
**Microbiome-based interventions**	Partially effective	Partially effective	Partially effective	Partially effective	Microbiome-based interventions act ecologically rather than at the level of the single-cell resistance determinant. They do not reverse hydrolysis, porin loss, efflux, or target modification directly, but may reduce intestinal reservoir burden, restore colonization resistance, lower recurrence risk, and reduce transmission of multidrug-resistant lineages.	Caballero-Flores et al. (2023); Davido et al. (2025)

Effectiveness assignments are intended as conceptual and relative ratings for Fig. 3A. These assignments are not prescriptive clinical recommendations and do not replace organism-specific susceptibility testing, infection-site considerations, severity assessment, or pharmacokinetic/pharmacodynamic optimization. The purpose of this table is to clarify the logic underlying Fig. 3A and to reduce overinterpretation of the effectiveness indicators as fixed clinical recommendations.

[a]Effective indicates that the strategy directly neutralizes, bypasses, or remains mechanistically orthogonal to the indexed resistance determinant when that mechanism is the dominant driver of failure. [b]Partially effective indicates that benefit is context-dependent or primarily adjunctive, improving regimen robustness, bacterial burden, virulence, or ecological control without directly reversing the mechanism itself. [c]Ineffective indicates that no consistent direct benefit is expected when the indexed mechanism is dominant.

References

Bush K, Bradford PA. 2019. Interplay between beta-lactamases and new beta-lactamase inhibitors. *Nat Rev Microbiol*. 17: 295–306.

Caballero-Flores G, Pickard JM, Nunez G. 2023. Microbiota-mediated colonization resistance: Mechanisms and regulation. *Nat Rev Microbiol.* 21: 347–360.

Davido B, Merrick B, Kuijper E, Benech N, Biehl LM, et al. 2025. How can the gut microbiome be targeted to fight multidrug-resistant organisms? *Lancet Microbe.* 6: 101063.

Fleitas Martinez O, Cardoso MH, Ribeiro SM, Franco OL. 2019. Recent advances in anti-virulence therapeutic strategies with a focus on dismantling bacterial membrane microdomains, toxin neutralization, quorum-sensing interference and biofilm inhibition. *Front Cell Infect Microbiol.* 9: 74.

Krishnamoorthy G, Leus IV, Weeks JW, Wolloscheck D, Rybenkov VV, et al. 2017. Synergy between active efflux and outer membrane diffusion defines rules of antibiotic permeation into Gram-negative bacteria. *mBio.* 8: e01172-17.

Liao C, Huang X, Wang Q, Yao D, Lu W. 2022. Virulence factors of *Pseudomonas aeruginosa* and antivirulence strategies to combat its drug resistance. *Front Cell Infect Microbiol.* 12: 926758.

Ogawara H. 2021. Possible drugs for the treatment of bacterial infections in the future: Anti-virulence drugs. *J Antibiot (Tokyo).* 74: 24–41.

Paul M, Carrara E, Retamar P, Tangden T, Bitterman R, et al. 2022. European Society of Clinical Microbiology and Infectious Diseases (ESCMID) guidelines for the treatment of infections caused by multidrug-resistant Gram-negative bacilli (endorsed by European society of intensive care medicine). *Clin Microbiol Infect.* 28: 521–547.

Sawa T, Moriyama K, Kinoshita M. 2024. Current status of bacteriophage therapy for severe bacterial infections. *J Intensive Care.* 12: 44.

Tamma PD, Heil EL, Justo JA, Mathers AJ, Satlin MJ, et al. 2024. Infectious Diseases Society of America 2024 guidance on the treatment of antimicrobial-resistant Gram-negative infections. *Clin Infect Dis.* ciae403.

Uyttebroek S, Chen B, Onsea J, Ruythooren F, Debaveye Y, et al. 2022. Safety and efficacy of phage therapy in difficult-to-treat infections: A systematic review. *Lancet Infect Dis.* 22: e208–e220.
